# *Cyclospora cayetanensis* Infection in Developed Countries: Potential Endemic Foci?

**DOI:** 10.3390/microorganisms11030540

**Published:** 2023-02-21

**Authors:** Leonor Chacin-Bonilla, Monica Santin

**Affiliations:** 1 Instituto de Investigaciones Clinicas, Universidad del Zulia, Maracaibo 4001, Venezuela; 2 Environmental Microbial and Food Safety Laboratory, Agricultural Research Service, United States Department of Agriculture, Beltsville, MD 20705, USA

**Keywords:** *Cyclospora cayetanensis*, cyclosporiasis, developed countries, endemic foci

## Abstract

*Cyclospora cayetanensis* infection has emerged as a significant public health concern worldwide. Developed countries are generally considered non-endemic for infection. However, sporadic cases and non-travel-related outbreaks of *C. cayetanensis* infections associated with domestically grown produce are becoming more common in developed countries. *Cyclospora cayetanensis* has been detected in fresh produce, surface water, wastewater, irrigation water, and soil in these countries, suggesting that the parasite may be more common in areas with advanced sanitation than previously thought and illustrating the potential risk for exposure and indigenous/autochthonous infections. The evidence suggests the possibility of foci of endemicity in developed countries, particularly in communities where sanitary conditions are compromised, and raises transmission issues that require further research to better define the risks for infection, how widespread *C. cayetanensis* may be in these areas, and to guide interventions against this infection. The main purpose of the present opinion was to evaluate the presence of cyclosporiasis in developed countries, which is a very important and ongoing issue in food safety.

## 1. Introduction

*Cyclospora cayetanensis*, a human coccidian parasite, infects the epithelial cells of the small intestine, causing gastrointestinal disease. It is the only species of the genus *Cyclospora* known to infect humans. Cyclosporiasis has emerged as a significant public health concern worldwide. Transmission is via the fecal–oral route. Human hosts become infected with *C. cayetanensis* when they ingest food or water contaminated with sporulated oocysts [1]. In tropical and subtropical countries, cyclosporiasis is endemic; most symptomatic cases that include watery diarrhea, abdominal cramps, loss of appetite, nausea, and low-grade fever are seen in children, while most adults are asymptomatic. The risk factors, routes of spread, and other epidemiologic features of this infection in these areas remain poorly understood. Variables related to water, eating raw fruits/vegetables, contact with animals, agrarian activities, lack of hand washing, deficient sanitary facilities, and contact with soil have been linked with infection [2,3,4,5,6,7]. An association of cyclosporiasis with poverty and soil transmission was observed in two Venezuelan communities, reflecting a relationship between household socioeconomic status with cyclosporiasis [8,9]. Fresh produce, water, and soil can become contaminated with oocysts and have all been implicated as sources of human infection [1,4,5,6,7,8,9].

The detection of *C. cayetanensis* in fresh produce and environmental samples and linking cyclosporiasis cases to sources of infection continue to be a challenge. Future efforts should assess the sources, modes of spread, and environmental distribution of the parasite. However, one of the obstacles to detecting the parasite in fresh produce and environmental samples is the low numbers of oocysts present in naturally contaminated fresh produce and environment samples, meaning that methods used for clinical samples are not always extrapolatable for detection in those samples. In recent years, several molecular techniques that have high specificity and sensitivity, including quantitative PCRs (qPCR), have been developed for the detection of *C. cayetanensis* in fresh produce. Validated protocols for the molecular detection of *C. cayetanensis* oocysts from fresh produce and agricultural water are publicly available [10], and the method in produce was recently adapted, verified, and employed in a national produce survey in Canada [11]. However, there is still the need for confirmatory molecular epidemiological methods of those samples found positive for the parasite to further elucidate relationships between isolates recovered from clinical specimens and implicated foods [11]. A genotyping system for *C. cayetanensis* based on eight genetic markers has been applied to human clinical samples [12]; however, this approach has not yet been employed successfully for food samples, and additional markers are required to improve cluster detection [11]. Recently, a workflow was successfully developed to obtain complete mitochondrial genome sequences from produce samples spiked with low numbers of *C. cayetanensis* for the typing of isolates [13,14].

## 2. Cyclosporiasis Situation in Epidemic Areas: Potential for Endemic Foci

In the past, in developed countries, outbreaks and sporadic cases of cyclosporiasis were largely caused by contaminated fresh produce imported from endemic areas and by travel to endemic places. Numerous outbreaks in the USA and Canada with clinical cases in people of any age were documented, mostly associated with imported fresh produce [10]. In the USA, *C. cayetanensis* has been responsible for numerous high-profile outbreaks of foodborne disease since the mid-1990s, often affecting multiple states. Cases identified in the USA prior to 1995 were all thought to be linked to imported food or with people who had traveled to endemic areas [10]. At that time, several cases might have been associated with exposure to contaminated drinking or recreational water or to sewage since there were waterborne and sporadic reports of infection where no food source or history of international travel was implicated [15,16,17]. However, in all those studies, there was a lack of information about the patient’s food history; therefore, the role of food as a potential source of infection cannot be discarded.

The first reported outbreak of cyclosporiasis in the USA was in Chicago, possibly linked to a hospital water supply [18]. Similarly, in Europe and Australia, most of the reported cases of cyclosporiasis were linked to international travel to endemic areas. In addition, foodborne outbreaks have also been reported [4,10,19]. For example, imported sugar snap peas from Guatemala were the suspected vehicle in a foodborne outbreak of *C. cayetanensis* in Stockholm (Sweden) based on information obtained from laboratory-confirmed *C. cayetanensis* in patients [19].

In recent years, sporadic and non-travel-related outbreaks of *C. cayetanensis* infection are becoming more common in developed countries. Domestically acquired outbreaks have been reported in Canada every spring or summer since 2013 [20]. In the USA, during the period of 2004–2009, 37.8% (70/185) of the cases were classified as domestically acquired [21]. Between 2000 and 2017, there were only 17/39 foodborne outbreaks of cyclosporiasis with a confirmed or suspected source [22]; from 2018 to 2022, thousands of laboratory-confirmed cases of cyclosporiasis in people who had no recent history of international travel before illness onset were reported in the USA [23]. Annual summertime outbreaks have become a regular phenomenon in the USA, and cases have been linked to both imported and locally grown fresh produce [24]. Importantly, the parasite has been detected in domestically produced foods in recent years [25]. The USA Food and Drug Administration recently reported *C. cayetanensis* contamination in domestically grown cilantro and romaine lettuce, which represents the first and second time that *C. cayetanensis* has been identified in produce grown in the USA. The importance of better defining risks for infection and how widespread *C. cayetanensis* may be in this country is underscored, and prevention, response, and a research action plan for this parasite were released [26]. To adopt a method of coordinated surveillance and investigation of cyclosporiasis outbreaks in Canada, the BAM Chapter 19b method, released by the U.S. Food and Drug Administration for the detection of *C. cayetanensis* in fresh produce in 2017 [27], was adapted, verified, and implement for routine surveillance in the Canadian Food Inspection Agency for the testing of fresh leafy greens, herbs, and berries for *C. cayetanensis* [11]. Using this method, a Canadian national survey on fresh produce detected *C. cayetanensis* in five samples (two berries, two herbs, and one leafy green), representing 0.28% of the tested survey samples. It is worth mentioning that three out of the five positive samples were fresh produce (spinach, cilantro, and blueberries) grown in the USA [11]. 

During non-outbreak periods, the rate of cyclosporiasis in the general population of North America and the United Kingdom was less than 0.5% between 1992 and 1995 [28]. In Sweden, in patients with gastroenteritis, the parasite was only detected in 2/714 (0.28%) fecal samples by microscopy and real-time PCR [29]. The incidence and suspected origin of those infections were unknown. Because the infection is not subject to notification in Sweden, the incidence may be most likely underestimated in this country. 

In Europe, the sources of a foodborne outbreak in Germany were epidemiologically traced to lettuce and herbs from Germany, France, and Italy; the contamination of food crops could have happened by agricultural workers without access to adequate sanitary facilities [30]. In Madrid, Spain, *C. cayetanensis* oocysts were detected in 9% of drinking, recreational, and wastewater samples; the annual prevalence of the parasite was 16.1% (9/56) in raw sewage and 10.7% (6/56) in treated sewage samples from conventional wastewater treatment plants, and 2% in four river basins [31]. In Italy, 30% (3/10) of train tap water samples were *C. cayetanensis*-positive; this elevated proportion of positive samples is a cause of concern considering the high oocyst viability and low infectious dose of *C. cayetanensis* oocysts required to start an infection [32]. In Apulia, Southern Italy, the parasite was identified in 15.5% of several environmental matrices, including treated wastewater (21.3% of 94 samples), well water (6.2% of 16), soil (11.8% of 51), and vegetables (12.2% of 49) [33]. Additionally, *Cyclospora* spp. has been previously reported in bivalve shellfish, often eaten raw, in Tunisia, Egypt, and Turkey [34,35,36]. *Cyclospora cayetanensis* was found in 35 mussels (*Mytilus galloprovincialis*), the most consumed shellfish species in Turkey, during a study that examined 795 mussels obtained at sites located on the west coast of Turkey [34]. *Cyclospora* spp. was also detected in gandofli (*Caelatura Iaronia pruneri*) obtained from local markets in Alexandria, Egypt [35]. *Cyclospora cayetanensis* was detected in wild clams (*Ruditapes decussatus*) collected along the Tunisian coasts [36]. A more recent study in Italy has identified *C. cayetanensis* in 36.4% of the Atlantic blue crab (*Callinectes sapidus*) examined from the Lesina Lagoon in the Mediterranean Sea [37]. The presence of *C. cayetanensis* in bivalves and in crabs suggests the contamination of the marine ecosystem. 

The presence of *Cyclospora* spp. has been reported in different water sources, such as irrigation water, wastewater, sewage, or surface water in the USA. In Southern Arizona, 19% (9/48) of wastewater samples from two plants were positive for *C. cayetanensis;* the oocysts were detected from both influent and effluent samples [38]. In Yuma, Arizona, and the Upper Rio Grande Valley, Texas, the parasite was found in raw sewage, treated wastewater, and irrigation waters in the produce-harvesting areas [39]. In the Chicago metropolitan area, 61 environmental samples were investigated for the presence of *C. cayetanensis* oocysts in three combined sewer outfalls systems [40]. There were 21 *C. cayetanensis*-positive samples: 13 from wildlife feces, 7 from soil, and 1 from water, giving the impression that the parasite may be more frequent in the USA than formerly considered and could be transferred by wildlife as paratenic hosts. The parasite was also detected in surface water samples in the Chesapeake and Ohio Canal (C&O Canal) in Maryland [41,42]. In Florida, cyclosporiasis was linked to gardening and working with soil [43]. These findings suggest a potential incidence of infection among the multiple settings studied and that farm workers could be at risk. 

The presence of *C. cayetanensis* in developed countries in produce, water, and soil, even in areas with advanced sanitation practices, illustrates the possibility of risk of exposure and potential endemic foci in these areas, making it difficult to avoid the protozoan from becoming endemic if the climate allows survival throughout the year. However, in developed countries, there are fewer chances for environmental contamination with the parasite. In these areas, imported produce may produce outbreaks of disease, but advanced sanitation and wastewater management systems should usually be enough to remove *C. cayetanensis* oocysts, mitigating transmission [40]. Therefore, it is expected that in developed countries, human waste would seldom come into contact with the environment, and *C. cayetanensis* would be unable to develop and infect another host. Thus, these places are generally considered non-endemic for infection [4,10]. However, even in highly developed countries, such as the USA, small and rural communities still have aging or inadequate wastewater treatment systems or do not have access to basic wastewater services [44]. Nowadays, there is mounting evidence showing some risk of exposure in developed areas with potential indigenous/autochthonous infections within the USA and other developed countries. The possibility of foci of low-level endemicity has already been considered in the USA [45] and in Italy, where autochthonous cases of the disease have been reported [46]. However, more studies are necessary as it has become clear that endemic cyclosporiasis is no longer just a threat to individuals living in developing countries with poor sanitation. 

We know little about how social inequality may mediate patterns of human exposure and cyclosporiasis, even in developed countries. The burden of neglected tropical diseases (NTDs) is largely concentrated in low- and lower-middle-income countries and is caused by climatic factors in combination with poverty-associated factors that favor the spread of the diseases. There is considerable evidence that socioeconomically disadvantaged groups have higher infection rates [47,48,49]. In high-income countries, it should be realized that there can be large within-country differences in income and health. In these areas, the actual burden of NTDs is arguably still concentrated in the poorest subgroups. Epidemiological evidence suggests that low socioeconomic conditions may augment the human risk of cyclosporiasis [8,9]; cross-sectional studies in Venezuela found individuals residing in extreme poverty had a higher prevalence of protozoan infections [50,51]. 

In the USA, the effects of family wealth on cryptosporidiosis risk have also been demonstrated; Hispanics and African Americans have greater odds of infection, and the risk is higher in families where income is low and close to the poverty threshold, for non-white and foreign-born persons [52]. These results highlight the potential links between social marginalization and *Cryptosporidium* infection in a developed country, as non-white and low-income immigrant and migrant populations are more prone to live in economically poor neighborhoods and have reduced access to resources [53,54]. These people may therefore be more likely to lack access to sanitation or educational resources for avoiding parasite exposure and be prone to live in physically impaired or remote environments. Individuals of all socioeconomic strata can acquire cyclosporiasis; however, these reports suggest that living in physically impaired environments could increase exposure to contaminated environmental matrices and influence patterns of infection risk so that the possibility of endemic foci in these surroundings exists. 

Social inequality could mediate patterns of human exposure and cyclosporiasis. Health inequities occur in developed countries among agricultural workers, and it has been shown that the physical health of migrant and seasonal farmworkers is worse than the general population due to occupational hazards as well as deficient living conditions [55]. Demographic data in the USA show that migrant farmworkers leave their homes every year to work in the fields in the USA, return to their countries and come back again the next year and are likely to amplify exposure to *C. cayetanensis* oocysts through contact with contaminated environmental vehicles. Most migrant farm workers live in substandard conditions, and nearly two-thirds of them live in poverty [56,57]. Racial residential segregation is a fundamental cause of differences in health status because it shapes socioeconomic conditions for non-white populations at the individual, household, neighborhood, and community levels, can create social and physical risks in residential environments that adversely affect health [53,54], and could support infectious diseases such as cyclosporiasis. Most fruits and vegetables produced in developed countries are harvested by hand, and the post-harvest handling of fresh produce also provides numerous opportunities for human contact. Therefore, due to the important role that farmworkers play in food safety, it is necessary that programs addressing farmworkers’ health are put in place and include advocating for good hygienic practices to reduce the risk of the transmission of foodborne parasites from agricultural commodities to consumers [58]. The recent unprecedented flow of people around the world and shifts in demographic trends, particularly in the USA, are factors that determine the introduction and favor the transmission and dissemination of parasites, including *C. cayetanensis*. Global warming is also a cause for concern as it may facilitate the survival of *Cyclospora* spp. and other pathogens over the winter, possibly resulting in endemic foci outside the tropics/subtropics [59]. Under likely climate change scenarios, it can be predicted that changes in the physical characteristics of the environment will lead to a global increase in entero-pathogens [60]. These factors make parasitic infections an even greater potential issue in developed countries. 

## 3. Conclusions

In conclusion, the evidence suggests that *C. cayetanensis* infection may be more common than currently acknowledged in developed countries. The likelihood of foci of endemicity, most likely in poor communities, raises transmission issues that require further research to better define the sources of infection, spread routes, and environmental distribution of *C. cayetanensis*. That information is critical to guide intervention actions to prevent transmission. Additional research studies are needed to identify any potential endemic foci. Those studies will require a multidisciplinary approach to include the sampling of a variety of potentially contaminated environmental sources, such as produce, water, soil, and animals.

## Data Availability

Not applicable.
